# Population-based incidence and mortality of community-acquired pneumonia in Germany

**DOI:** 10.1371/journal.pone.0253118

**Published:** 2021-06-15

**Authors:** Christian Theilacker, Ralf Sprenger, Friedhelm Leverkus, Jochen Walker, Dennis Häckl, Christof von Eiff, Julia Schiffner-Rohe

**Affiliations:** 1 Pfizer Pharma GmbH, Berlin, Germany; 2 Pfizer Deutschland GmbH, Berlin, Germany; 3 InGef–Institute for Applied Health Research Berlin, Berlin, Germany; 4 WIG2 GmbH, Leipzig, Germany; University of Notre Dame Australia, AUSTRALIA

## Abstract

**Background:**

Little information on the current burden of community-acquired pneumonia (CAP) in adults in Germany is available.

**Methods:**

We conducted a retrospective cohort study using a representative healthcare claims database of approx. 4 million adults to estimate the incidence rates (IR) and associated mortality of CAP in 2015. IR and mortality were stratified by treatment setting, age group, and risk group status. A pneumonia coded in the primary diagnosis position or in the second diagnosis position with another pneumonia-related condition coded in the primary position was used as the base cases definition for the study. Sensitivity analyses using broader and more restrictive case definitions were also performed.

**Results:**

The overall IR of CAP in adults ≥18 years was 1,054 cases per 100,000 person-years of observation. In adults aged 16 to 59 years, IR for overall CAP, hospitalized CAP and outpatient CAP was 551, 96 and 466 (with a hospitalization rate of 17%). In adults aged ≥60 years, the respective IR were 2,032, 1,061 and 1,053 (with a hospitalization rate of 52%). If any pneumonia coded in the primary or secondary diagnosis position was considered for hospitalized patients, the IR increased 1.5-fold to 1,560 in the elderly ≥60 years. The incidence of CAP hospitalizations was substantially higher in adults ≥18 years with at-risk conditions and high-risk conditions (IR of 608 and 1,552, respectively), compared to adults without underlying risk conditions (IR 108). High mortality of hospitalized CAP in adults ≥18 was observed in-hospital (18.5%), at 30 days (22.9%) and at one-year (44.5%) after CAP onset. Mortality was more than double in older adults in comparison to younger patients.

**Conclusion:**

CAP burden in older adults and individuals with underlying risk conditions was high. Maximizing uptake of existing vaccines for respiratory diseases may help to mitigate the disease burden, especially in times of strained healthcare resources.

## Introduction

Community-acquired pneumonia (CAP) causes high rates of hospitalizations and is associated with substantial premature death and a high clinical and economic burden for healthcare systems in Europe [1,2]. For Germany, an annual incidence of hospitalization for CAP of approximately 970 per 100,000 inhabitants was estimated in 2010/2011 [3]. However, studies previously performed to analyze the frequency of CAP in Germany are outdated, investigated restricted age groups or excluded certain risk groups, most notably immunocompromised patients [3–5]. CAP risk increases with age as well as with underlying chronic medical conditions, such as chronic obstructive pulmonary disease (COPD), asthma, diabetes mellitus, congestive heart failure (CHF), immunosuppression, cancer, and smoking [4,6]. Also, aging itself increases the risk for pneumonia and pneumococcal disease due to immunosenescence, multimorbidity, and frailty [7,8] with CAP rates in older adults being not dissimilar from respective rates in younger adults with immunocompromising conditions [9]. Therefore, the German Standing Committee on Vaccination (Ständige Impfkommission, STIKO) recommends for example a pneumococcal vaccination (PV) for adults aged 60 years and older and for adults with defined risk factors [10]. However, only 31% of eligible elderly Germans have received a lifetime pneumococcal vaccine dose [11]. In a cohort study in patients with a newly diagnosed “high-risk” condition between January 2013 and December 2014, pneumococcal vaccination rate within two years after first diagnosis of the risk condition was only 4.4%, with highest vaccination rates among patients starting immunosuppressive treatments for rheumatoid arthritis (11.5%) and patients with a newly diagnosed HIV infection (9.9%) [12]. From a public health perspective, optimized uptake of existing vaccines for respiratory diseases also helps to mitigate the annual burden of disease from influenza and pneumococcal pneumonia and as a consequence maximizes the availability of limited health care resources such as intensive care unit (ICU) beds in respiratory virus pandemics such as SARS-CoV-2 [13–15]. In this regard, the CAPITA trial demonstrated that vaccination of older adults with the 13-valent pneumococcal conjugate vaccine also reduced the number of days spend in hospital or in the ICU by prevention of episodes of clinical pneumonia [13]. The number needed to vaccinate (NNV, number of persons needed to vaccinate to prevent one additional outcome of interest) in this trial was 22 to prevent a single hospital day for clinical CAP and 145 to prevent a day on the intensive care unit.

In an aging society like in Germany an increasing pneumonia burden can be expected over time and updated estimates of CAP incidence rates and related outcomes are needed. Therefore, the aim of this study was to give a recent and robust estimate of the population-based incidence rate of CAP and mortality of incident-hospitalized CAP cases.

## Material and methods

### Data source

This study was conducted based on the InGef (former HRI) database with anonymized data from approximately 6.7 million individuals in Germany insured in one of approximately 70 German statutory health insurance providers (SHIs) currently contributing data to this database (mainly company or guild health insurances) [4,16]. For this study, a representative sample of the German SHI population regarding age and sex (approximately five percent of the German population) was created. This sample size comprised of 4,221,022 patients. The database includes demographic information (e.g., age and sex), information on outpatient healthcare services, data related to hospital treatment, including admission and discharge date, diagnoses, operations and interventions (OPS codes) as well as prescription and dispensation of reimbursed remedies and aids. All diagnoses in the database were coded according to the German modification of the 10^th^ revision of the International Classification of Diseases (ICD-10 GM) [17]. Data contributing to the InGef database are stored at a specialized data center owned by SHIs providing data warehouse services. In the data center (acting as a trust center), data with respect to individual insured members, health care providers (e.g. physicians, practices, hospitals, pharmacies), and the respective SHI are anonymized. All data were analyzed by InGef-Staff based on the study protocol and only aggregated data were provided.

### Ethics approval and consent to participate

All patient-level data in the InGef research database are de-identified to comply with German data protection regulations. Use of the study database for health services research is therefore fully compliant with German federal law and, accordingly, institutional review board/ethical approval and informed consent of the patient was not needed.

### Study design and population

We conducted a retrospective cohort study of the healthcare claims database described above. First, we estimated the population-based incidence rate of CAP per 100,000 person years in 2015. Further, a cohort of patients with an incident diagnosis of CAP in 2015 was identified to calculate in-hospital mortality, all-cause 30-day mortality and the all-cause one-year mortality after CAP diagnosis.

In order to estimate the incidence rate of CAP, a population-based cohort was created in 2015. Cohort entry was January 1^st^, 2015, if all inclusion criteria were fulfilled. Subjects were eligible to enter the population-based cohort, if they were (i) 16 years of age or older in the observation period (study period), (ii) continuously insured in the respective year or until death, (iii) had at least one year of continuous insurance prior to the study year (baseline period) and (iv) did not have a hospital or outpatient diagnosis of pneumonia (see case definition below) in the last quarter of the baseline period. The baseline period was necessary to exclude prevalent cases of CAP and to assess diagnoses for the risk-group classification. Patients were included in the study cohort that accumulated person-years of observation from cohort entry date until the earliest date among the occurrence of CAP or censoring (occurring at death or December 31, 2015).

Patients were eligible for the CAP cohort, if they (i) were 16 years of age or older in the observation period (study period), (ii) had at least one diagnosis for CAP (see case definition below) in 2015, (iii) no diagnosis of any pneumonia in the last quarter of 2014, (iv) continuous insurance for at least 365 days or until death after the incident CAP diagnosis (observation period). Cohort entry was the hospital admission date or the date of the antibiotic treatment of CAP in the ambulatory setting (index date, see case definition below). Patients were followed until death or 365 days after cohort entry, whichever occurred first.

### Study endpoints

Incident CAP was defined as the first CAP event in the observation period and reported overall and stratified by treatment setting. For patients with an outpatient diagnosis of CAP and a subsequent CAP hospitalization in the following 60 days, the case was classified as hospitalized CAP. CAP episodes that occurred more than 60 days apart and were treated in different settings (hospital and outpatient) were considered separate events.

For the base case definition, we defined hospitalized CAP as a primary hospital diagnosis of pneumonia (ICD-10 codes A48.1, B01.2, J10.0, J11.0, J12-J18, J69.0, J85.1) OR a secondary CAP hospital diagnosis in combination with a hospital admission diagnosis of pneumonia OR a secondary hospital diagnosis of pneumonia in combination with a primary hospital diagnosis of sepsis (ICD-10 GM codes A40.3, A40.8, A40.9, A41) [3]. The date of hospital admission was defined as the index date of hospitalized CAP. Patients with hospital-acquired pneumonia (secondary code of U69.00 according to the German ICD-10-GM 2019 and a hospitalization of > 2 days), and patients discharged from the hospital within 7 days prior to the index date were excluded.

The base case definition for outpatient CAP was a verified ambulatory diagnosis of pneumonia as defined above in combination with a prescription for an antibiotic (Anatomical Therapeutic Classification (ATC) codes J01AA*, J01CA* (excl. J01CA08), J01CE*, J01CR*, J01DB*, J01DC*, J01DD*, J01DE*, J01DH*, J01EE*, J01FA*, J01MA*, J05AB*). The date of the antibiotic prescription was defined as the index date of outpatient CAP.

To analyze the impact of our case definition on the measured CAP incidence, we performed several sensitivity analyses (for detailed case definitions see S6 Table). As a broader case definition for hospitalized CAP, cases with either a primary or secondary hospital diagnosis for pneumonia were included (sensitivity analysis 1). In a second sensitivity analysis for hospitalized cases, patients with pneumonia according to the base case definition OR an ICD-10 code for acute lower respiratory tract infections (LRTI) in the primary coding position were included (sensitivity analysis 2). In the outpatient setting, a sensitivity analysis was performed that included patients meeting either the base case definition or patients with an ICD-10 code for LRTI (sensitivity analysis 1, S6 Table). For a second sensitivity analysis the case definition required the diagnoses of CAP as defined for the primary analysis plus a radiologic investigation (chest X-ray, CT scan or magnetic resonance imaging [MRI]) in the same quarter (sensitivity analysis 2).

Further study outcomes included mortality after incident CAP due to any cause in-hospital, at 30-days or 365-days.

### Risk factors for CAP

Underlying chronic medical conditions predisposing to CAP were defined according to the STIKO indication group classification for pneumococcal vaccination as at-risk conditions [18]. At-risk conditions included chronic heart disease, chronic pulmonary disease (including asthma), diabetes mellitus and neurological disorders. Immunocompromising conditions and anatomical or foreign body associated risk factors were considered high-risk condition as well as functional or anatomic asplenia, sickle cell diseases and other hemoglobinopathies, chronic severe liver disease, HIV infection, chronic renal failure or dialysis, malignant neoplasms/radiation therapy/cytotoxic chemotherapy, autoimmune diseases, immunosuppressive treatment, solid organ or stem cell transplantation, congenital immunodeficiency, and neutropenia/agranulocytosis. Risk conditions were identified based on hospital and verified outpatient diagnoses as well as OPS codes subdivided into at-risk conditions and high-risk conditions (for details see S7 and S8 Tables). If patients had both at-risk conditions and high-risk conditions, they were classified as high-risk. Patients without any at-risk or high-risk condition were assigned to the low-risk group.

### Statistical analysis

#### Calculation of CAP incidence rate and predisposing factors for CAP

The incidence rate of CAP per 100,000 person-years of observation (PYO) was calculated by dividing the absolute number of cases by the amount of cumulative person time in the cohort, overall and stratified by age-groups (≥ 18 years, 16–59 years and ≥60 years). Further strata included risk status (low-risk, at-risk, high-risk) and individual risk factors. For descriptive purposes, 95% confidence intervals (CI) were provided for the incidence rates of each outcome assuming a Poisson distribution. Incidence rate ratios (IRR) and 95% confidence intervals were calculated for disease episodes in persons with at-risk and high risk conditions versus their age-matched counterparts without underlying risk conditions (low risk). To compare the impact of age versus immunocompromising conditions on the CAP incidence rates, we calculated IRRs of hospitalized CAP in adults aged ≥60 years in comparison to younger adults aged 16–59 years with high-risk conditions.

#### Calculation of mortality after CAP

Mortality in-hospital, at 30-days, and at one year after CAP diagnosis in 2015 were calculated based on the CAP cohort. Mortality rates were calculated overall and further stratified by age group and risk group status as defined above. For descriptive purposes, 95% confidence intervals (CI) were provided for mortality rates.

## Results

### Characteristics of study population

**Fig 1:**
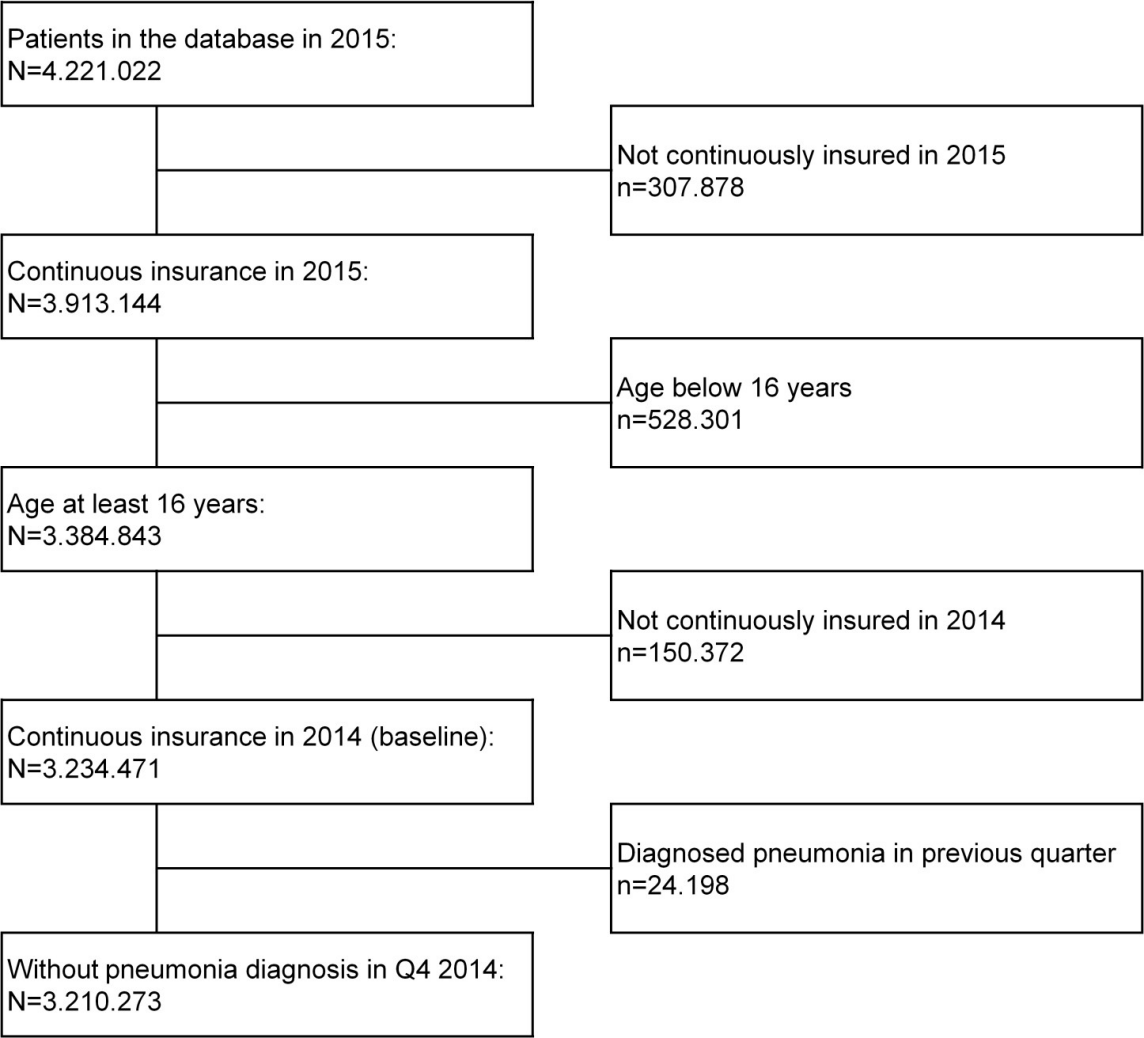
Study flow.

The population-based cohort for the year 2015 comprised approximately 3.21 million individuals 16 years and older (Fig 1). Of those, 66.5% were between 16 and 59 years of age, and 33.5% were 60 years or older. 74.2% of adults aged 16–59 years had no medical conditions predisposing to pneumococcal disease (“low-risk”), whereas 19.6% had one at-risk condition (and no high-risk conditions), and 6.2% had a high-risk condition (S4 Table). In this age group, the most frequent medical conditions were chronic pulmonary disease (13.8%), chronic heart disease (3.5%) and diabetes mellitus (3.2%). The prevalence of predisposing conditions increased with age and 36.9% and 26.6% of adults aged ≥60 years had at-risk and high-risk conditions, respectively. The most frequent at-risk conditions in older adults were chronic heart disease (18.5%), diabetes mellitus (16.4%) and chronic pulmonary disease (14.4%). The most frequently observed high-risk conditions were solid and hematologic malignancies and chronic renal failure, both mainly contributed by the elderly, as well as autoimmune diseases (S5 Table).

### Pneumonia incidence rates

Over the one-year study period, a total of 33,134 first CAP episodes as defined by the base case definition occurred within a total follow up time of 3,184,720 person-years of observation (PYO) in persons 16 years and older. The IR of overall combined outpatient and hospitalized CAP in individuals ≥18 years of age in 2015 was 1,054 cases per 100,000 PYO (Table 1). The overall CAP incidence rate in adults aged ≥60 years was almost fourfold higher compared to the age group 16–59 years (2,032 versus 551). CAP incidence varied widely by age group and risk group status (Table 1). In low-risk adults, the incidence rate increased from 386 in persons aged 16–59 years to 789 in those aged ≥60 years. Corresponding IR in at-risk adults were 937 and 2,191, and in high-risk adults corresponding rates were 1,310 and 3,577. In individuals with at-risk conditions, CAP rates were 2.40–3.45 times higher compared to low-risk individuals of the same age group depending on age groups and treatment setting (see corresponding IRR in Table 1). Similarly, CAP incidence rates in adults with high-risk conditions were 2.81 times to 6.68 times in comparison with low-risk adults. CAP incidence rates among individuals with defined at-risk or high-risk conditions varied substantially by risks conditions and are reported in S1 and S2 Tables. To better understand the impact of age relative to immunocompromising conditions on pneumonia rates, we compared incidence rates among hospitalized adults aged ≥ 60 years with rates in younger immunocompromised adults (S3 Table). Incidence rates ratios of CAP among adults ≥ 60 years overall and immunocompetent adults ≥ 60 years (i.e. low risk/at-risk status), relative to younger, immunocompromised persons (i.e. 16 to 59 years with high risk conditions) were 2.6, and 1.7, respectively. Also, incidence rate ratios > 1 were also observed for adults ≥ 60 years when compared to CAP rates among adults aged 16–59 years with the most prevalent immunocompromising conditions (S3 Table). Hospitalization rates for CAP were dependent on age with a lower rate for CAP in patients aged 16–59 years (17%), compared to patients aged ≥60 years (52%).

**Table 1:** Incidence rates and incidence rate ratios of CAP in 2015 according to case definitions for hospitalized and outpatient CAP.

Measure	Number of PYO			Pneumonia Incidence Rates and Rate Ratios					
	16–59 years	≥ 60 years	≥ 18 years	16–59 years		≥ 60 years		≥ 18 years	
				IR per 100K (95% CI)	Rate Ratio (95% CI)	IR per 100K (95% CI)	Rate Ratio (95% CI)	IR per 100K (95% CI)	Rate Ratio (95% CI)
**All Pneumonia**									
Overall pneumonia incidence (base case)	2,131,780	1,052,940	3,111,713	551 (541–561)		2,032 (2,004–2,059)		1,054 (1,043–1,066)	
Low-risk	1,583,038	390,033	1,912,705	386 (376–396)	Reference	789 (762–818)	Reference	469 (460–479)	Reference
At-risk	416,786	389,557	795,225	937 (908–967)	2.43 (2.33–2.53)	2,191 (2145–2238)	2.78 (2.66–2.89)	1,553 (1,525–1,580)	3.31 (3.22–3.40)
High-risk	131,956	273,350	403,783	1,310 (1249–1374)	3.40 (3.22–3.58)	3,577 (3,507–3,649)	4.53 (4.35–4.72)	2,844 (2,792–2,896)	6.06 (5.90–6.23)
**Hospitalized Pneumonia**									
Overall pneumonia incidence (base case)	2,131,780	1,052,940	3,111,713	96 (92–100)		1,061 (1,041–1,081)		423 (416–430)	
Low-risk	1,583,038	390,033	1,912,705	55 (51–59)	Reference	314 (296–332)	Reference	108 (103–113)	Reference
At-risk	416,786	389,557	795,225	151 (140–164)	2.75 (2.48–3.05)	1,083 (1,050–1,116)	3.45 (3.24–3.68)	608 (591–625)	5.63 (5.35–5.93)
High-risk	131,956	273,350	403,783	414 (380–450)	7.51 (6.75–8.36)	2,096 (2,042–2,151)	6.68 (6.28–7.11)	1,552 (1,514–1,591)	14.37 (13.68–15.11)
1° and 2° CAP codes (sensitivity analysis 1)	2,131,501	1,050,996	3,109,483	125 (120–129)		1,560 (1,536–1,584)		611 (603–620)	
CAP and LRTI (sensitivity analysis 2)	2,131,145	1,050,838	3,108,989	145 (140–150)		1,400 (1,378–1,423)		570 (562–579)	
**Outpatient Pneumonia**									
Overall pneumonia incidence (base case)	2,126,850	1,051,231	3,105,209	466 (457–476)		1,053 (1,033–1,072)		667 (658–676)	
Low-risk	1,580,266	389,417	1,909,414	337 (328–346)	Reference	496 (474–518)	Reference	370 (361–379)	Reference
At-risk	415,125	388,715	792,758	807 (780–835)	2.40 (2.30–2.50)	1,203 (1,169–1,238)	2.43 (2.30–2.56)	1,003 (981–1,025)	2.71 (2.63–2.80)
High-risk	131,459	273,099	403,037	947 (895–1001)	2.81 (2.64–2.99)	1,632 (1,585–1,681)	3.29 (3.12–3.47)	1,411 (1,375–1,448)	3.82 (3.68–3.95)
CAP plus radiology (sensitivity analysis 2)	2,130,296	1,055,049	3,112,337	199 (193–205)		413 (401–425)		274 (269–280)	
CAP and LRTI (sensitivity analysis 1)	2,063,451	1,022,114	3,014,502	5,408 (5,376–5,439)		5,605 (5,559–5,651)		5,495 (5,469–5,522)	

CAP: Community-acquired pneumonia; LRTI: Lower respiratory tract infection, PYO: Person-years of observation.

Incidence rates are shown per 100.000 person-years of observation (95% confidence interval). Patients with no at-risk or high-risk condition were classified as low-risk status; patients with ≥ 1 at-risk condition and no high-risk condition were classified as at-risk; patients with any high-risk condition were classified as high-risk status.

We performed sensitivity analyses to explore the impact of variation of the case definitions on CAP incidences rate measures. When both primary and secondary pneumonia codes were considered for the case definition of hospitalized CAP, incidence rate estimates increased 1.3-fold to 125 in adults aged 16 to 59 years and 1.47-fold to 1,560 in adults ≥ 60 years (Table 1). Similarly, inclusion of both CAP and LRTI codes in the case definition led to a substantial increase in the incidence estimates (sensitivity analysis 2 in hospitalized patients and sensitivity analysis 1 in outpatients, Table 1). This was most pronounced in the outpatient setting, where in adults ≥ 18 years the incidence rate increased 8.2-fold to 5,495 compared to the base case definition (Table 1). In contrast, if a radiologic investigation in the same quarter was required to diagnose CAP in the outpatient setting (sensitivity analysis 2), the CAP incidence estimate in adults ≥ 18 years decreased 59% to 274 (Table 1).

### Mortality rate of CAP

Among 11,598 CAP patients aged ≥18 that met the base case definition of hospitalized CAP, mortality in-hospital, at 30 days and one-year were 18.5%, 22.9% and 44.5% (Table 2). Pneumonia mortality substantially increased with age: mortality in-hospital, at 30-day and at one-year were twice as high in older adults ≥60 years of age (19.8%, 24.5%, and. 47.4%) compared to younger patients (8.4%, 10.0% and 21.3%). In adults ≥60 years of age, underlying at-risk conditions had no significant impact on mortality measures compared to CAP episodes that occurred in low-risk individuals of that age group (Table 2). Mortality rates of older adults with high-risk conditions were moderately higher compared to mortality rates in patients belonging to the at-risk or low-risk group. In younger adults with hospitalized CAP, by contrast, underling risk factors substantially increased the mortality of hospitalized CAP (Table 2). In this age group, 30-day mortality rates in low-risk, at-risk and high-risk persons were 2.8%, 8.6% and 20.9%, with similar patterns observed for the in-hospital and 1-year mortality.

**Table 2:** Mortality of hospitalized CAP according to case definition and risk group status.

Measure	No. Pneumonia cases			Mortality after pneumonia hospitalization (%)		
	16–59 years	≥ 60 years	≥ 18 years	16–59 years	≥ 60 years	≥ 18 years
**In-hospital Mortality**						
Overall mortality (base case)	1,294	10,330	11,598	8.4 (6.9–10.0)	19.8 (19.0–20.5)	18.5 (17.8–19.2)
Mortality low-risk status	496	1,094	1,577	2.0 (1.0–3.7)	17.6 (15.4–20.0)	12.9 (11.3–14.6)
Mortality at-risk status	420	3,855	4,266	8.1 (5.7–11.1)	17.5 (16.3–18.8)	16.6 (15.5–17.8)
Mortality high-risk status	378	5,381	5,755	16.9 (13.3–21.1)	21.8 (20.7–22.9)	21.5 (20.4–22.6)
Mortality 1° and 2° CAP codes (sensitivity analysis 1)	1,814	15,878	17,667	10.9 (9.5–12.4)	20.0 (19.3–20.6)	19.0 (18.5–19.6)
Mortality CAP and LRTI (sensitivity analysis 2)	1,874	13,518	15,357	4.7 (3.8–5.8)	14.0 (13.4–14.6)	12.9 (12.3–13.4)
**30-day Mortality**						
Overall Mortality (base case)	1,294	10,330	11,598	10.0 (8.4–11.7)	24.5 (23.7–25.4)	22.9 (22.2–23.7)
Mortality low-risk status	496	1,094	1,577	2.8 (1.6–4.7)	22.0 (19.6–24.6)	16.2 (14.4–18.1)
Mortality at-risk status	420	3,855	4,266	8.6 (6.1–11.7)	22.0 (20.7–23.4)	20.7 (19.5–22.0)
Mortality high-risk status	378	5,381	5,755	20.9 (16.9–25.4)	26.8 (25.7–28.0)	26.5 (25.3–27.6)
Mortality 1° and 2° CAP codes (sensitivity analysis 1)	1,814	15,878	17,667	11.4 (9.9–12.9)	23.9 (23.2–24.5)	22.6 (22.0–23.2)
Mortality CAP and LRTI (sensitivity analysis 2)	1,874	13,518	15,357	7.2 (6.0–8.4)	20.4 (19.7–21.1)	18.8 (18.2–19.5)
**1-year Mortality**						
Overall Mortality (base case)	1,294	10,330	11,598	21.3 (19.1–23.6)	47.4 (46.4–48.3)	44.5 (43.6–45.4)
Mortality low-risk status	496	1,094	1,577	5.9 (4.0–8.3)	38.9 (36.0–41.9)	28.8 (26.6–31.1)
Mortality at-risk status	420	3,855	4,266	15.0 (11.7–18.8)	41.4 (39.8–42.9)	38.8 (37.3–40.3)
Mortality high-risk status	378	5,381	5,755	48.4 (43.3–53.6)	53.4 (52.0–54.7)	53.1 (51.8–54.4)
Mortality 1° and 2° CAP codes (sensitivity analysis 1)	1,814	15,878	17,667	24.2 (22.2–26.2)	48.7 (47.9–49.5)	46.2 (45.5–46.9)
Mortality CAP and LRTI (sensitivity analysis 2)	1,874	13,518	15,357	16.8 (15.1–18.5)	42.0 (41.2–42.8)	39.0 (38.2–39.8)

CAP: Community-acquired pneumonia; LRTI: Lower respiratory tract infection.

Mortality due to any cause in-hospital, at 30 days and at 365 days after CAP diagnosis are reported. Patients with no at-risk or high-risk condition were classified as low-risk status; patients with ≥ 1 at-risk condition and no high-risk condition were classified as at-risk; patients with any high-risk condition were classified as high-risk status.

No significant differences in mortality rates between our base case definition and CAP defined as any primary or secondary CAP diagnosis code was observed (sensitivity analysis 1, Table 2). In contrast, the mortality rates were generally lower, if CAP was defined as either a CAP diagnosis according to the base case definition or LRTI as the principal diagnosis code.

## Discussion

In the current analyses, we have estimated the population-based incidence of CAP in Germany, using a large health claims database that is representative of the overall German population. CAP incidence in German adults remains high with an overall rate of 1,054 per 100,000 person-years of observation. CAP rates were approximately fourfold higher in older adults compared to younger individuals (2,032 versus 551) and CAP incidence increased with the presence of underlying at-risk or high-risk conditions. Of note, CAP incidence estimates for CAP hospitalizations in older adults were approximately 1.5-fold higher (1,560 vs. 1,061) if a case definition was used that included patients with any primary or secondary diagnosis code. With approximately 1 in 4 patients aged ≥60 years dying within 30 days and 1 in 2 patients dying within one year after CAP hospitalization, CAP was associated with a high mortality rate in older adults in Germany. Although mortality in younger patients hospitalized for CAP was substantially lower, still 10% died within 30 days and 21% died within one year, with substantially higher mortality rates in patients with at-risk or high-risk conditions.

CAP incidence for Germany has been reported by several previous publications [3–5,19]. Due to differences in study designs and populations, case definitions and study periods, however, the results are difficult to compare. Schnoor et al. measured annual CAP incidence in the German city of Luebeck in 2004 using several study designs [5]. Depending on the methodology used, CAP incidences between 370 and 1,010 per 100,000 in adults aged ≥18 were estimated. Ewig et al. used CAP hospitalization data from a national clinical audit program (Institute for Quality Assurance and Transparency in Healthcare, IQTIG), which records pneumonia codes in the primary diagnosis position and in non-immunocompromised patients only [19]. For the year 2005/2006, the study reported an incidence of 296 and 765 per 100,00 for patients aged ≥18 and ≥60, respectively. A retrospective cohort study based on health claims data used a case definition comparable to our base case definition and analyzed CAP incidence and mortality rate for the year 2010/2011 [3]. Like our results, the study estimated an overall CAP incidence of 970 per 100,000 person-years aged ≥18 years. The case definition used in the study by Pelton et al., on the other hand, resembled our definition for sensitivity analysis 1, i.e. any CAP diagnosis code in the primary or secondary coding position [4]. Compared to our results, this study, however, found higher CAP incidences of 1,439, 3,547 and 5,851 per 100,000 person years in adults aged ≥60 with low-risk, at-risk and high-risk conditions, respectively. Finally, Germany’s national clinical audit program (IQTIG), reported a total of 289,020 CAP hospitalizations for the year 2018, which equates to an incidence of 97 and 1,012 per 100,000 for adults aged ≥18 and ≥60, if German population census data are used for the denominator [20]. As noted above, IQTIG only includes non-immunocompromised patients in their survey and only uses primary diagnoses codes for its reporting. Three recent literature reviews have summarized CAP incidence in Europe, the US and globally [2,21,22]. CAP incidence rates across Europe varied widely between 68–7,000 per 100,000 [2]. With a median incidence rate of hospitalized CAP in older adults of 1,830 per 100,000 patient years CAP incidence rates in the United States seem to be higher compared to our estimates for Germany [21]. Finally, the meta-estimate for hospitalized CAP IR for industrialized nations was 680 per 100,000 PYO for older adults aged 65–74 years, 1,640 in those 75–84 years and 3,460 in those aged ≥85 years [22].

While long-term mortality rates after CAP are heterogenous, with mortality rates varying between 10% and > 80% [23], several studies have also reported mortality rates after CAP hospitalizations in Germany with findings similar to our study [3,20,24,25]. The observed one-year mortality rate of 44.5% for hospitalized CAP in adults ≥ 18 years measured in our study was higher compared to a recent study from the US where 29% of CAP patient died within one year after pneumonia [26].

Noteworthy, Kolditz et al. reported 30 day mortality for hospitalized CAP of 21.9% for adults aged ≥18 years, a rate very comparable to our result for this age group (22.9%) [3]. Mortality was also analyzed in a small retrospective single-center cohort for the years 2005 to 2009 [24,25]. Of 559 hospitalized patients analyzed, 10.9% died within 30 days, 21% died within a year. However, because of the single center design, the external validity of this study is limited. Furthermore, the analysis for the one-year mortality only included patients that were still alive at day 30 after CAP, and thus underestimated the true one-year mortality. In-hospital mortality in Germany’s national clinical audit (IQTIG) for the year 2018 was 13.6% and therefore somewhat lower than reported by us (18.5%). The most likely explanation for this discrepancy is the exclusion from immunocompromised patients from the clinical audit program, which according to our findings carry a higher mortality than CAP patients with low-risk or at-risk conditions (21.5% versus 12.8% and 16.6%, respectively).

In our study, we have explored the impact of CAP case definitions on IR estimates. Our findings underscore the importance of choosing the most appropriate case definition for the measurement of CAP incidence in studies using health claims data. Numerous studies have demonstrated that pneumonia may lead to the exacerbation underlying comorbidities, such as acute cardiac events, asthma or COPD [27–32]. As CAP is the antecedent and not direct cause of hospitalization in these patients, it is likely that CAP is coded in a non-primary position. Also, pneumonia in older people may present with absence of fever, the paucity or absence of cough, and changes in mental status (delirium), also increasing the likelihood of CAP not diagnosed in the primary coding position [33]. Furthermore, the diagnostic sensitivity of admission chest x-rays in the diagnosis of CAP is limited, adding further to its diagnostic misclassification [34–36]. Taken together, studies that define pneumonia only as pneumonia coded in the primary diagnosis position or–as done in our base case definition—as pneumonia coded in the primary diagnosis position or in the second diagnosis position with another pneumonia-related condition coded in the primary position may substantially underestimate the full disease burden. Similar observations have been made by a recent systematic review of CAP incidence rates in the US, which found that rates of CAP in studies that did not consider non-primary diagnosis codes may underestimate the true disease burden [21]. Also, a validation study from Australia found that only 50.8% of ICD-10 pneumonia codes J10-J18 were coded in the primary diagnostic position, but 82% of cases were documented in the first four positions and 95% in the first eight positions [37].

Pneumonia diagnosis in the outpatient setting is even more challenging. This is due to large overlap of the clinical signs and symptoms of CAP with other LRTIs and the low availability of radiologic imaging in the outpatient setting. A large European study that analyzed 2,810 patients that presented with acute cough to their general practitioner, only 29% of patients with radiologically confirmed CAP were diagnosed as CAP based on their medical history and physical examination [38]. Based on these findings, it is justified to assume that a proportion of outpatient LRTI, as included in one of our sensitivity analyses, do represent true pneumonias that were misclassified as LRTI. Our results also suggest that including a radiologic imaging study in the case definition for outpatient CAP likely leads to substantial underestimation of CAP in the ambulatory setting.

In our study, we also explored the impact of advanced age relative to immunocompromising conditions on pneumonia risk and documented significantly higher incidence rates in immunocompetent adults ≥ 60 years of age compared to younger, immunocompromised adults. This comparison could be informative to pneumococcal vaccine recommendations based on the rationale that if the risks are similar in these groups, a coherent pneumococcal immunization strategy would treat them the same based on comparable immunocompromised state with respect to pneumococcal disease.

Our study was not without limitations. Although the InGef database covers nearly four million insured members of SHIs all over Germany, representativeness for the whole SHI population can only be guaranteed regarding age and sex. Therefore, the CAP incidence and the other outcomes obtained in this study may not be fully generalizable to the whole German population. Further, the IR of CAP as well as mortality after CAP diagnosis are estimates and are not standardized for age, sex and other risk factors. Finally, hospital administrative data show only modest sensitivity for detecting CAP if compared to prospective clinical studies as the gold standard, may leave a quarter of pneumonia cases undetected [39].

In conclusion, our study showed that the overall CAP burden in German adults remains high. With an incidence between 1,061 and 1,560 per 100,000 person-years, this is most notable for CAP hospitalizations of older adults. In this context, effective prevention strategies for CAP are needed, especially in the elderly, and may have a high potential to prevent premature deaths as well as to reduce the CAP-related economic burden for the German healthcare system. Further studies may be needed to validate burden estimates from health claims data against gold standard methods, such as population-based prospective, active pneumonia surveillance.

## Supporting information

S1 Table Incidence rate of CAP (base case definition) in patients with at-risk conditions stratified by treatment setting. (PDF)

S2 Table Incidence rate of CAP (base case definition) in patients with high-risk conditions stratified by treatment setting. (PDF)

S3 Table Comparison of hospitalized CAP incidence rate in adults ≥ 60 years of age versus adults aged 16–59 years with underlying immunocompromising (high-risk) conditions. (PDF)

S4 Table Prevalence of underlying risk conditions, number of individuals per risk group status and total number CAP cases per risk group status according to the base case definition of CAP. (PDF)

S5 Table Prevalence of underlying high-risk conditions, number of individuals per risk group status and total number CAP cases per risk group status according to the base case definition of CAP. (PDF)

S6 Table Case definitions used in the sensitivity analyses. (PDF)

S7 Table Definition of at-risk conditions for CAP. (PDF)

S8 Table Definition of high-risk conditions for CAP. (PDF)

## Abbreviations

ATC: Anatomical therapeutic classification
CAP: Community-acquired pneumonia
CHF: Congestive heart failure
CI: Confidence interval
COPD: Chronic obstructive pulmonary disease
ICD-10 GM: International Classification of Diseases, 10th Revision, German Modification
ICU: Intensive Care Unit
InGef: Institute for Applied Health Research Berlin
IQTiG: Institute for Quality Assurance and Transparency in Healthcare
IR: Incidence rate
LRTI: Lower respiratory tract infection
MRI: Magnetic resonance imaging
NNV: Number needed to vaccinate
OPS: Operationen- und Prozedurenschlüssel, an addition to the German ICD-10 GM version
PV: Pneumococcal vaccination
PYO: Person-Years of Observation
SHI: Statutory health insurance provider
STIKO: German Standing Committee on Vaccination (Ständige Impfkommission)
