# Supplementary material for: Population-based incidence and mortality of community-acquired pneumonia in Germany
Source: PLoS One. 2021 Jun 15;16(6):e0253118. doi: 10.1371/journal.pone.0253118 (PMC8205119; doi:10.1371/journal.pone.0253118)
Supplement: S1 Table — (PDF) [file pone.0253118.s001.pdf]

Supplementary Table 1 Incidence Rate of CAP (base case definition) in patients with at-risk conditions stratified by treatment setting.

	Number of PYO			Pneumonia incidence per 100,000 PYO					
				16 – 59 years		≥ 60 years		≥ 18 years	
	16 – 59 years	≥ 60 years	≥ 18 years	IR	95% CI	IR	95% CI	IR	95% CI
**All pneumonia**									
Chronic heart disease	74,109	194,592	267,806	977	907 – 1,051	2,520	2,450 – 2,592	2,097	2,043 – 2,153
Chronic pulmonary disease	294,985	152,158	437,826	1024	988 – 1,061	2,979	2,893 – 3,067	1,708	1,669 – 1,747
Diabetes mellitus	67,750	173,277	240,658	970	897 – 1,047	2,072	2,005 – 2,141	1,764	1,711 -1,818
Neurological disorders	28,923	26,550	54,542	1303	1175 – 1,442	4,847	4,586 -5,120	3,020	2,876 – 3,169
**Hospitalized Pneumonia**									
Chronic heart disease	74,109	194,592	267,806	186	156 - 220	1,300	1,250 – 1,352	995	957 – 1,033
Chronic pulmonary disease	294,985	152,158	437,826	147	134 - 162	1,345	1,288 – 1,405	564	542 - 586
Diabetes mellitus	67,750	173,277	240,658	196	164 - 233	1,068	1,020 – 1,118	824	788 - 861
Neurological disorders	28,923	26,550	54,542	522	442 - 612	3,126	2,917 – 3,346	1,788	1,677 – 1,903
**Outpatient Pneumonia**									
Chronic heart disease	73,820	194,204	267,133	818	754 - 886	1334	1283 - 1387	1193	1152 - 1236
Chronic pulmonary disease	293,646	151,533	435,890	898	864 - 933	1775	1709 - 1844	1208	1175 - 1241
Diabetes mellitus	67,504	173,000	240,135	801	735 - 872	1097	1048 - 1148	1015	975 - 1056
Neurological disorders	28,853	26,616	54,541	856	753 - 970	2,014	1,847 – 2,192	1,415	1,317 – 1,519

CI: Confidence interval; IR: Incidence rate; YO: Person-years of observation
