# Supplementary material for: Population-based incidence and mortality of community-acquired pneumonia in Germany
Source: PLoS One. 2021 Jun 15;16(6):e0253118. doi: 10.1371/journal.pone.0253118 (PMC8205119; doi:10.1371/journal.pone.0253118)
Supplement: S2 Table — (PDF) [file pone.0253118.s002.pdf]

	Number of PYO			Pneumonia incidence per 100,000 PYO					
				16 – 59 years		≥ 60 years		≥ 18 years	
	16 – 59 years	≥ 60 years	≥ 18 years	IR	95% CI	IR	95% CI	IR	95% CI
**All Pneumonia**									
Chronic severe liver disease	7632	12392	20001	1572	(1304 - 1880)	3567	(3242 - 3915)	2805	(2578 - 3047)
Autoimmune disease	29606	38043	67463	1206	(1084 - 1338)	2952	(2782 - 3130)	2188	(2078 - 2302)
Functional or anatomic asplenia	4068	3436	7414	1967	(1559 - 2448)	6344	(5530 - 7245)	3979	(3538 - 4460)
HIV	2410	511	2911	1660	(1186 - 2260)	4309	(2701 - 6524)	2130	(1633 - 2731)
Chronic renal failure	21736	110510	132079	1882	(1704 - 2073)	4950	(4819 - 5083)	4450	(4337 - 4565)
Solid and hematologic malignancies	52257	142194	194181	1407	(1307 - 1512)	3278	(3184 - 3373)	2776	(2703 - 2851)
Solid organ transplantation	1666	1412	3050	6484	(5319 - 7828)	9563	(8018 - 11319)	7935	(6967 - 9001)
Congenital immunodeficiency	14398	5785	19473	1327	(1145 - 1529)	4201	(3689 - 4763)	2162	(1960 - 2379)
Immunosuppressive treatment	15062	12501	27421	1613	(1417 - 1829)	4136	(3787 - 4508)	2764	(2571 - 2968)
Diseases of white blood cells	4492	4559	8967	1558	(1215 - 1969)	5001	(4373 - 5695)	3301	(2936 - 3699)
**Hospitalized Pneumonia**									
Chronic severe liver disease	7632	12392	20001	550	(397 - 744)	2130	(1881 - 2404)	1530	(1363 - 1711)
Autoimmune disease	29606	38043	67463	270	(214 - 336)	1446	(1327 - 1572)	931	(859 - 1007)
Functional or anatomic asplenia	4068	3436	7414	1008	(723 - 1367)	4307	(3641 - 5060)	2522	(2174 - 2911)
HIV	2410	511	2911	415	(199 - 763)	2546	(1356 - 4355)	790	(501 - 1186)
Chronic renal failure	21736	110510	132079	713	(605 - 835)	3124	(3020 - 3230)	2731	(2643 - 2822)
Solid and hematologic malignancies	52257	142194	194181	547	(486 - 615)	1902	(1831 - 1975)	1537	(1483 - 1593)
Solid organ transplantation	1666	1412	3050	4022	(3117 - 5108)	5880	(4683 - 7289)	4886	(4133 - 5736)
Congenital immunodeficiency	14398	5785	19473	278	(198 - 378)	2195	(1830 - 2612)	847	(723 - 987)
Immunosuppressive treatment	15062	125011	27421	611	(492 - 749)	2216	(1963 - 2493)	1342	(1208 - 1486)
Diseases of white blood cells	4492	4559	8967	890	(636 - 1213)	3378	(2866 - 3956)	2141	(1849 - 2466)

Supplementary Table 2 Incidence Rate of CAP (base case definition) in patients with high-risk conditions stratified by treatment setting.

Supplementary Table 2 continued Incidence Rate of CAP (base case definition) in patients with high-risk conditions stratified by treatment setting.

	Number of PYO			Pneumonia incidence per 100,000 PYO					
				16 – 59 years		≥ 60 years		≥ 18 years	
	16 – 59 years	≥ 60 years	≥ 18 years	IR	95% CI	IR	95% CI	IR	95% CI
**Outpatient Pneumonia**									
Chronic severe liver disease	7600	12381	19958	1066	(846 - 1325)	1607	(1392 - 1847)	1398	(1239 - 1572)
Autoimmune disease	29478	37916	67207	977	(867 - 1097)	1643	(1517 - 1777)	1353	(1266 - 1443)
Functional or anatomic asplenia	4060	3453	7422	985	(704 - 1342)	2462	(1966 - 3044)	1671	(1390 - 1992)
HIV	2397	510	2897	1293	(879 - 1836)	2156	(1076 - 3857)	1450	(1045 - 1960)
Chronic renal failure	21649	110631	132113	1247	(1103 - 1405)	2037	(1953 - 2122)	1908	(1834 - 1984)
Solid and hematologic malignancies	52094	142015	193837	914	(833 - 1000)	1508	(1445 - 1574)	1351	(1299 - 1403)
Solid organ transplantation	1671	1422	3065	2872	(2117 - 3808)	4291	(3282 - 5511)	3556	(2920 - 4290)
Congenital immunodeficiency	14319	5764	19378	1090	(925 - 1275)	2255	(1884 - 2678)	1419	(1256 - 1597)
Immunosuppressive treatment	15007	12465	27330	1086	(926 - 1266)	2150	(1900 - 2423)	1573	(1428 - 1729)
Diseases of white blood cells	4491	4558	8963	735	(506 - 1032)	1931	(1549 - 2379)	1350	(1120 - 1613)
