# Supplementary material for: Population-based incidence and mortality of community-acquired pneumonia in Germany
Source: PLoS One. 2021 Jun 15;16(6):e0253118. doi: 10.1371/journal.pone.0253118 (PMC8205119; doi:10.1371/journal.pone.0253118)
Supplement: S3 Table — (PDF) [file pone.0253118.s003.pdf]

Supplementary Table 3 Comparison of hospitalized CAP incidence rate in adults ≥ 60 years of age versus adults aged 16 – 59 years with underlying immunocompromising (high-risk) conditions. For number of number of individuals with risk condition, risk group or risk conditions prevalence, corresponding patient years of observation, and number of CAP cases, please refer to Table 1, Supplementary Table 2, Supplementary Table 4 and Supplementary Table 5.

Age Group and Risk Group Status	Hospitalized CAP Incidence Rate per 100, 000 (95% Confidence Interval)	Incidence Rate Ratio (95% Confidence Interval)			
		≥ 60 Yrs. Overall vs. 16 – 59 Yrs. High Risk	≥ 60 Yrs. At-risk vs. 16 – 59 Yrs. High Risk	60 Yrs. Low risk/at-risk vs. 16 – 59 Yrs. High Risk	60 Yrs. Low risk vs. 16 – 59 Yrs. High Risk
**Age 60+ years**					
Overall	1061 (1041 – 1081)	Comparator			
At-risk conditions	1083 (1050 – 1116)		Comparator		
Low risk or at-risk conditions	698 (679 – 717)			Comparator	
Low risk conditions	314 (295 – 332)				Comparator
**Age 16 to 59 years**					
**High risk overall**	414 (380 – 450)	2.56 (2.35 - 2.80)	2.62 (2.39 - 2.87)	1.69 (1.54 - 1.84)	0.76 (0.68 - 0.84)
Autoimmune disease	270 (214 – 336)	3.93 (3.15 - 4.95)	4.01 (3.21 - 5.06)	2.58 (2.07 - 3.26)	1.16 (0.93 - 1.47)
Chronic renal failure	713 (605 – 835)	1.49 (1.27 - 1.75)	1.52 (1.29 - 1.79)	0.98 (0.83 - 1.16)	0.44 (0.37 - 0.52)
Solid & hematologic malignancies	547 (486 – 615)	1.94 (1.72 - 2.19)	1.98 (1.75 - 2.24)	1.28 (1.13 - 1.44)	0.57 (0.50 - 0.65)
Immunosuppressive treatment	611 (492 – 749)	1.74 (1.41 - 2.16)	1.77 (1.44 - 2.20)	1.14 (0.93 -1.42)	0.51 (0.42 - 0.64)
