# Supplementary material for: Population-based incidence and mortality of community-acquired pneumonia in Germany
Source: PLoS One. 2021 Jun 15;16(6):e0253118. doi: 10.1371/journal.pone.0253118 (PMC8205119; doi:10.1371/journal.pone.0253118)
Supplement: S4 Table — (PDF) [file pone.0253118.s004.pdf]

Supplementary Table 4 Prevalence of underlying risk conditions, number of individuals per risk group status and total number CAP cases per risk group status according to the base case definition of CAP.

Risk Group Status	Prevalence	N	Pneumonia Cases		
			All	Hospitalized	Outpatient
**16 – 59 years**					
Low-Risk conditions overall	74.2%	1,583,937	6,108	872	5,321
At-Risk conditions overall	19.6%	417,438	3,906	631	3,352
Chronic heart disease	3.5%	74,295	724	138	604
Chronic pulmonary disease	13.8%	295,378	3,020	434	2637
Diabetes mellitus	3.2%	67,908	657	133	541
Neurological disorders	1.4%	29,080	377	151	247
High-Risk conditions overall	6.2%	133,052	1,729	546	1245
**≥ 60 years**					
Low-Risk conditions overall	36.5%	392,729	3,078	1,223	1,931
At-Risk conditions overall	36.9%	396,923	8,535	4,217	4,676
Chronic heart disease	18.5%	199,210	4,904	2,530	2,591
Chronic pulmonary disease	14.4%	155,266	4,533	2,047	2690
Diabetes mellitus	16.4%	176,609	3,591	1,851	1898
Neurological disorders	2.6%	279,63	1,287	830	536
High-Risk conditions overall	26.6%	286,194	9,778	5,729	4458
**≥ 18 years**					
Low-Risk conditions overall	61.1%	1,916,280	8,974	2,065	7,064
At-Risk conditions overall	25.6%	803,232	12,347	4,832	7,950
Chronic heart disease	8.7%	272,608	5,617	2,664	3,188
Chronic pulmonary disease	14.1%	441,318	7,477	2,468	5264
Diabetes mellitus	7.8%	244,147	4,245	1,983	2437
Neurological disorders	1.8%	56,108	1,647	975	772
High-Risk conditions overall	13.3%	417,716	11,483	6,266	5688
