# Supplementary material for: Population-based incidence and mortality of community-acquired pneumonia in Germany
Source: PLoS One. 2021 Jun 15;16(6):e0253118. doi: 10.1371/journal.pone.0253118 (PMC8205119; doi:10.1371/journal.pone.0253118)
Supplement: S5 Table — (PDF) [file pone.0253118.s005.pdf]

Supplementary Table 5 Prevalence of underlying high-risk conditions, number of individuals per risk group status and total number CAP cases per risk group status according to the base case definition of CAP.

Risk Group Stutus	Prevalence	N	Pneumonia Cases		
			All	Hospitalized	Outpatients
**16 – 59 years**					
Chronic severe liver disease	0.4%	7,773	120	42	81
Autoimmune disease	1.4%	29,687	357	80	288
Functional or anatomic asplenia	0.2%	4,152	80	41	40
HIV	0.1%	2,422	40	10	31
Chronic renal failure	1.0%	22,045	409	155	270
Solid and hematologic malignancies	2.5%	52,998	735	286	476
Solid organ transplantation	0.1%	1,744	108	67	48
Congenital immunodeficiency	0.7%	14,434	191	40	156
Immunosuppressive treatment	0.7%	15,157	243	92	163
Diseases of white blood cells	0.2%	4,591	70	40	33
**≥ 60 years**					
Chronic severe liver disease	1.2%	13,140	442	264	199
Autoimmune disease	3.6%	39,039	1,123	550	623
Functional or anatomic asplenia	0.4%	3,820	218	148	85
HIV	0.0%	528	22	13	11
Chronic renal failure	11.0%	118,289	5,470	3,452	2,253
Solid and hematologic malignancies	13.8%	148,826	4,661	2,704	2,142
Solid organ transplantation	0.1%	1,534	135	83	61
Congenital immunodeficiency	0.6%	5,972	243	127	130
Immunosuppressive treatment	1.2%	12,902	517	277	268
Diseases of white blood cells	0.5%	4,964	228	154	88
**≥ 18 years**					
Chronic severe liver disease	0.7%	20,890	561	306	279
Autoimmune disease	2.2%	68,538	1,476	628	909
Functional or anatomic asplenia	0.3%	7,880	295	187	124
HIV	0.1%	2,940	62	23	42
Chronic renal failure	4.5%	140,166	5,877	3,607	2,521
Solid and hematologic malignancies	6.4%	201,551	5,391	2,985	2,618
Solid organ transplantation	0.1%	3,250	242	149	109
Congenital immunodeficiency	0.6%	19,695	421	165	275
Immunosuppressive treatment	0.9%	27,916	758	368	430
Diseases of white blood cells	0.3%	9,469	296	192	121
