# Supplementary material for: Population-based incidence and mortality of community-acquired pneumonia in Germany
Source: PLoS One. 2021 Jun 15;16(6):e0253118. doi: 10.1371/journal.pone.0253118 (PMC8205119; doi:10.1371/journal.pone.0253118)
Supplement: S6 Table — (PDF) [file pone.0253118.s006.pdf]

**Supplementary Table 6** Case definitions used in the sensitivity analyses

| Definitions for sensitivity analyses | |
|---|---|
| **Hospitalized patients** | |
| **Sensitivity Analysis 1**<br><br>Pneumonia diagnosis code in primary or secondary coding positions | Patients with a primary or secondary hospital diagnosis of pneumonia (ICD-10 codes A48.1, B01.2, J10.0, J11.0, J12-J18, J69.0, J85.1).<br><br>Patients with (i) a secondary hospital diagnosis indicating hospital-acquired pneumonia (ICD-10 GM code U69.00) in a hospital stay with a duration of at least two days OR (ii) a hospital discharge in 7 days prior to the index date will not be considered as hospitalized CAP case.<br><br>The hospital admission date is defined as the index date for CAP |
| **Sensitivity Analysis 2**<br><br>Hospitalized CAP or acute infection of the lower respiratory tract<br><br>[CAP + LRTI] | Patients with a primary hospital diagnosis of pneumonia or acute infections of the lower respiratory tract (ICD-10 codes A48.1, B01.2, J09-J18, J20-J22 J69.0, J85.1) **OR** a secondary hospital diagnosis in combination with a hospital admission diagnosis of pneumonia or acute infections of the lower respiratory tract **OR** a secondary hospital diagnosis of pneumonia or acute infections of the lower respiratory tract in combination with a primary hospital diagnosis of sepsis (ICD-10 GM codes A40.3, A40.8, A40.9, A41).<br><br>Patients with (i) a secondary hospital diagnosis indicating hospital-acquired pneumonia (ICD-10 GM code U69.00) in a hospital stay with a duration of at least two days **OR** (ii) a hospital discharge in 7 days prior to the index date will not be considered as hospitalized CAP case<br><br>The hospital admission date is defined as the index date for CAP. |
| **Outpatient CAP** | |
| **Sensitivity Analysis 1**<br><br>Any CAP or LRTI diagnosis code | Patients with a verified ambulatory diagnosis of pneumonia or acute infections of the lower respiratory tract (ICD-10 codes J09.x-J18.x, J20.x-J22.x) in combination with a prescription for an antibiotic (ATC codes J01AA*, J01CA* (excl. J01CA08), J01CE*, J01CR*, J01DB*, J01DC*, J01DD*, J01DE*, J01DH*, J01EE*, J01FA*, J01MA*, J05AB*).<br><br>The date of the antibiotic prescription is defined as the index date for CAP. |
| **Sensitivity Analysis 2**<br><br>CAP diagnosis code in combination with radiological investigation | Patients with a verified ambulatory diagnosis of pneumonia in combination with a prescription for an antibiotic (ATC codes J01AA*, J01CA* (excl. J01CA08), J01CE*, J01CR*, J01DB*, J01DC*, J01DD*, J01DE*, J01DH*, J01EE*, J01FA*, J01MA*, J05AB*) **AND** at least one recorded EBM-code for a chest x-ray (EBM codes 34240, 34241, 34242), CT-scan (EBM-codes 34330) or MRI (EBM-codes 34430) in the same quarter.<br><br>The date of the antibiotic prescription is defined as the index date for CAP. |
