# Supplementary material for: Population-based incidence and mortality of community-acquired pneumonia in Germany
Source: PLoS One. 2021 Jun 15;16(6):e0253118. doi: 10.1371/journal.pone.0253118 (PMC8205119; doi:10.1371/journal.pone.0253118)
Supplement: S7 Table — (PDF) [file pone.0253118.s007.pdf]

Supplementary Table 7 Definition of at-risk conditions for CAP

Variable	Operational definition
Chronic heart disease	Patients with a primary or secondary hospital diagnosis or verified ambulatory diagnosis I05.x-I09.x, I11.x, I13.x, I20.x, I21.x, I22.x, I24.x, I25.1, I25.2, I27.x, I34.x-I39.x, I42.x, I50.x, I23.x, Q20.x-Q24.x, Q25.1
Chronic pulmonary disease (incl. asthma)	Patients with a primary or secondary hospital diagnosis or verified ambulatory diagnosis E84x, I278, I279, J40x-J44x, J47x, J60x-J65x, J684, J84x, J96x, P27x
Asthma bronchiale	Patients with a primary or secondary hospital diagnosis or verified ambulatory diagnosis E84.x, I27.8, I27.9, J40.x-J47.x, J60.x-J65.x, J68.4, J84.x, J96.x, P27.x
Diabetes mellitus	Patients with a primary or secondary hospital diagnosis or verified ambulatory diagnosis E10.x-E14.x, P70.1, T38.3, G59.0, G63.2
Neurological disorders	Patients with a primary or secondary hospital diagnosis or verified ambulatory diagnosis G041, G114, G240, G242, G40x, G41.x, G71.x, G72.x, G80.x, P90.x, R56.x, G81.x, G82.x, G83.0-G83.4, G83.9
