# Supplementary material for: Population-based incidence and mortality of community-acquired pneumonia in Germany
Source: PLoS One. 2021 Jun 15;16(6):e0253118. doi: 10.1371/journal.pone.0253118 (PMC8205119; doi:10.1371/journal.pone.0253118)
Supplement: S8 Table — (PDF) [file pone.0253118.s008.pdf]

Supplementary Table 8 Definition of high-risk conditions for CAP

Variable	Operational definition
Autoimmune disease	Patients with a primary or secondary hospital diagnosis or verified ambulatory diagnosis M05.x, M06.x, M32.x, K50.x
Functional or anatomic asplenia, sickle cell diseases and other hemoglobinopathies	Patients with a primary or secondary hospital diagnosis or verified ambulatory diagnosis Q89.0, Q89.3, D56.x, D57.x, D58.2, D60.x, D61.x, D73.0, D73.1, D73.8
HIV infection	Patients with a primary or secondary hospital diagnosis or verified ambulatory diagnosis B20.x-B23.x, B24.x, Z21.x
Chronic renal failure or dialysis	Patients with a primary or secondary hospital diagnosis or verified ambulatory diagnosis I12.0, I13.1, I13.2, N01.x, N03.x, N04x, N05.x, N18.x, N19.x, Q60.x, Z49.x, Z94.0, Z99.2 OR an OPS code (8853.x, 8854.x, 8855.x, 8857.x) or EBM code (13602, 13610, 13611, 40823-40828) in the year prior to cohort entry
Chronic severe liver disease	Patients with a primary or secondary hospital diagnosis or verified ambulatory diagnosis K70.x (excl. K70.0), K71.3-K71.8, K72.1-K72.9, K73.x, K74.x, K75.0, K75.1, K76.2-7, I85.0, I85.9
Solid organ or stem cell transplantation	Patients with a primary or secondary hospital diagnosis or verified ambulatory diagnosis Z940-Z944, Z9480, Z9481 or with an OPS code 5411.x, 8805.x, 5335.x, 5375.x, 5504.x, 5555.x
Congenital immunodeficiency	Patients with a primary or secondary hospital diagnosis or verified ambulatory diagnosis D80.x-D84.x, D89.x, D71.x
Neutropenia/agranulocytosis	Patients with a primary or secondary hospital diagnosis or verified ambulatory diagnosis D70.x
Immunosuppressive treatment	Patients with at least one prescription of a drug with the ATC code L04*
Malignant neoplasm/radiation therapy/cytotoxic chemotherapy	Patients with a hospital diagnosis or verified ambulatory diagnosis (ICD-10 GM code) C00.x- C97.x (excl. C44.*), Z51.0, Z51.1 OR an OPS code 852x.x (radiation therapy) OR an OPS code 8542.x, 8543.x, 8544.0, 8544.1 (cytotoxic chemotherapy) OR with a prescription of a drug with the ATC code L01* (cytotoxic chemotherapy)
